# The shifting of buffer crop repertoires in pre-industrial north-eastern Europe

**DOI:** 10.1038/s41598-025-87792-0

**Published:** 2025-01-29

**Authors:** Meiirzhan Abdrakhmanov, Michael Kempf, Ruta Karaliute, Piotr Guzowski, Rimvydas Lauzikas, Margaux L. C. Depaermentier, Radosław Poniat, Giedre Motuzaite Matuzeviciute

**Affiliations:** 1 Department of Archaeology, Faculty of History, Vilnius University, Universiteto St. 7, Vilnius, 01513 Lithuania; 2 Quaternary Geology, Department of Environmental Sciences, University of Basel, Bernoullistrasse 32, Basel, 4056 Switzerland; 3 Department of Geography, University of Cambridge, Cambridge, UK; 4 Lithuanian Institute of History, Tilto St. 17, Vilnius, 01101 Lithuania; 5 Faculty of Communication, Vilnius University, Saulėtekio ave. 9, Vilnius, 10222 Lithuania; 6 Faculty of History, University of Bialystok, Plac NZS 1, Bialystok, 15-420 Poland

**Keywords:** Archaeobotany, Climate change, Buffer crops, Little ice age, Northern latitudes, Documentary sources, Palaeoclimate, Plant sciences

## Abstract

This study explores how major climatic shifts, together with socioeconomic factors over the past two millennia, influenced buffer crop selection, focusing on five crops: rye, millet, buckwheat, oat, and hemp. For this study, we analyzed archaeobotanical data from 135 archaeological contexts and historical data from 242 manor inventories across the northeastern Baltic region, spanning the period from 100 to 1800 AD. Our findings revealed that rye remained a main staple crop throughout the studied periods reflecting environmental adaptation to northern latitudes. The drought-tolerant and thermophilic millet crop exhibited resilience during the adverse dry climatic conditions of the Medieval Climatic Anomaly while showing a significant decline during the Little Ice Age. During the period of post-1500 AD, a significant shift towards cold-resilient summer crops such as buckwheat and hemp is recorded. This study enhances our understanding of how historical agricultural systems responded to both socioeconomic factors and climatic change in northern latitudes, offering notable potential solutions for modern agricultural practices in the face of future climate variability trends.

**Supplementary Information:**

The online version contains supplementary material available at 10.1038/s41598-025-87792-0.

## Introduction

The past two millennia have seen significant climatic fluctuations in Europe, including phases of increased warming and cooling or prolonged droughts and wet spells. These changes are well documented through various proxies such as tree-rings, speleothems, and pollen records^1–5^. Some climatic periods such as the Medieval Climate Anomaly (MCA) and the Little Ice Age (LIA) are characterized by particularly distinct environmental conditions. These climatic shifts have had direct effects on agricultural productivity and crop choices, with diversification often seen as a response to deteriorating conditions^6–8^.

In regions prone to climatic variability such as the temperate zones of northeastern Europe, certain crops functioned as buffer crops. Buffer crops mitigate risks associated with adverse climatic and environmental stress, providing stability to agricultural systems during periods of instability^9–11^. Archaeobotanical studies of temperate northeastern Europe have shown that barley and glume wheats constitute the main staple foods across several millennia, while the use of other crops such as rye, millet, oat, hemp and buckwheat fluctuated across time^12^. Such crops, however, have played an important role in the past, offering resilience against prolonged drought periods, generally cold climates, or poor soil quality. These crops, through their adaptive nature, served as a form of agricultural insurance when more climate-sensitive staples, such as bread wheat, struggled to thrive.

Food systems in geographically marginal regions, such as mountain highlands or temperate regions of northeastern Europe, are particularly sensitive to climatic fluctuations. These marginal areas often face harsher environmental conditions, making them more vulnerable to variations in temperature and precipitation, which can directly impact crop yields and agricultural sustainability^13–16^. It is important to draw the attention to the fact, that the Eastern Baltic region above 54° northern latitude has long been constituted the historical northern limit for successful cultivation of broomcorn millet (hereafter – millet), a thermophilic and globally important to food security crop, which has been an important staple food here since the Bronze Age^17^. Yet, the cultivation of millet has varied and then declined in past millennia, raising the question whether this decline was linked with decreases in temperature. If so, millet could be a viable option for future agriculture in connection with rising global temperatures.

In the southern regions of Eurasia and Africa, multiple examples have identified the shifts of crop repertoires as response to environmental change^7,9,18^. However, there is limited research on how the populations of temperate regions in northeastern Europe adapted their agricultural practices to local climatic fluctuations. This region, with its future potential to become a significant agricultural area under changing global climates, offers valuable insights into historical agroeconomic strategies.

**Fig. 1:**
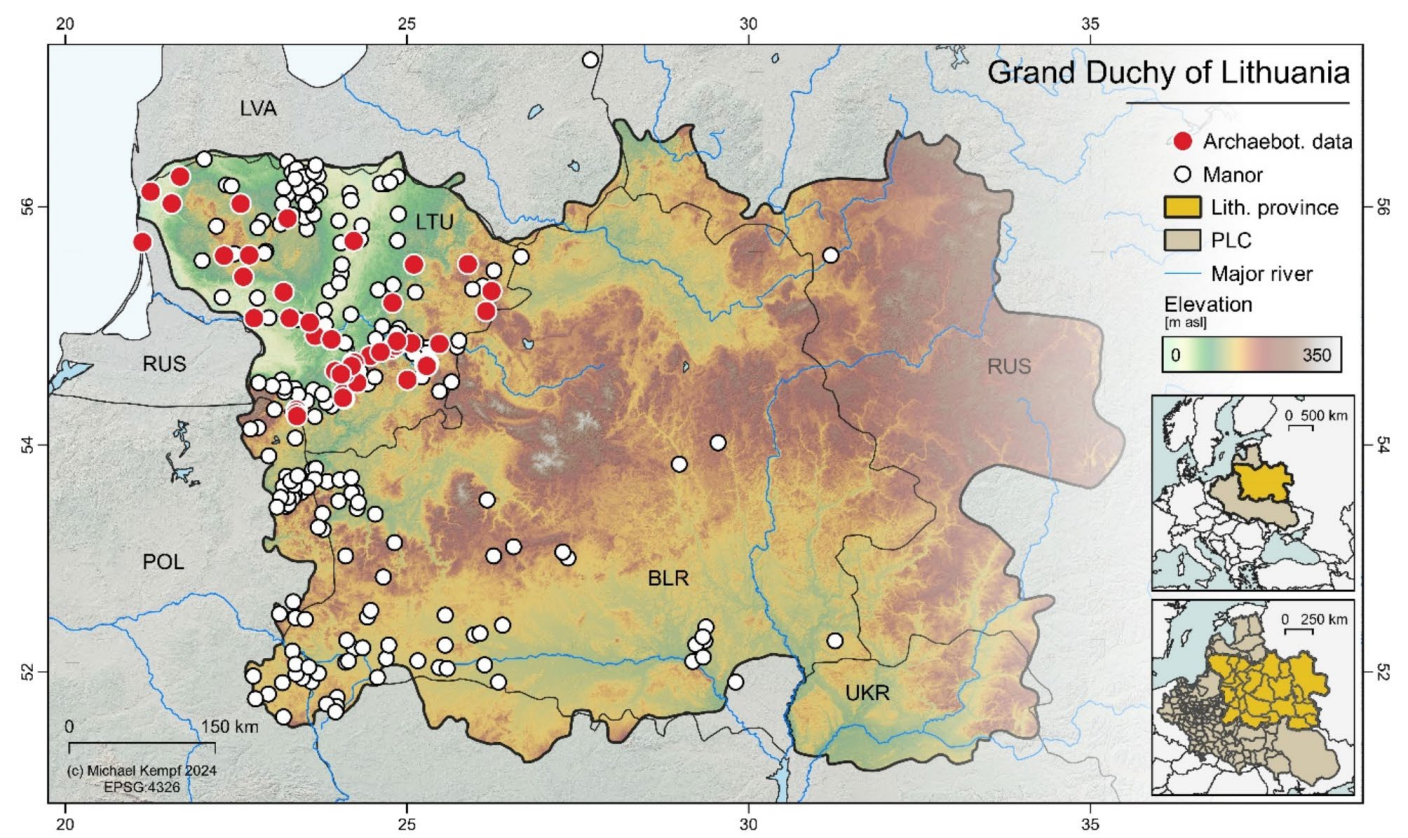
Geographical location of the historical Grand Duchy of Lithuania within the European context (PLC, Polish-Lithuanian Commonwealth). The map shows the study area (Lithuanian Province of the PLC), including the locations of manors where historical data (1500–1800 AD) was analysed and the archaeobotanical sample sites. Digital elevation model derived from SRTM90 v4.1^22^. This map is produced using QGIS 3.10.12 (QGIS Geographic Information System. QGIS Association. http://www.qgis.org (2024)).

This study focuses on the combined analysis of both archaeobotanical and historical datasets. The archaeobotanical data was obtained from 135 contexts across 98 archaeological sites. The historical analysis mainly sourced from archives includes 242 descriptions of crop structures on royal and noble manors from the Early Modern period (1500–1800 AD) in the Grand Duchy of Lithuania (Fig. 1). We analyzed the distribution of crops such as rye, oat, millet, buckwheat, and hemp from 100 AD to 1800 AD, with previously published climatic data from the Eastern Baltic region. By analyzing the shifts in crop distribution over the past two millennia, we aim to understand the agricultural responses to climatic transitions and their implications for urban development and resilience.

## Results

### Archaeobotanical and climatic analysis

**Fig. 2:**
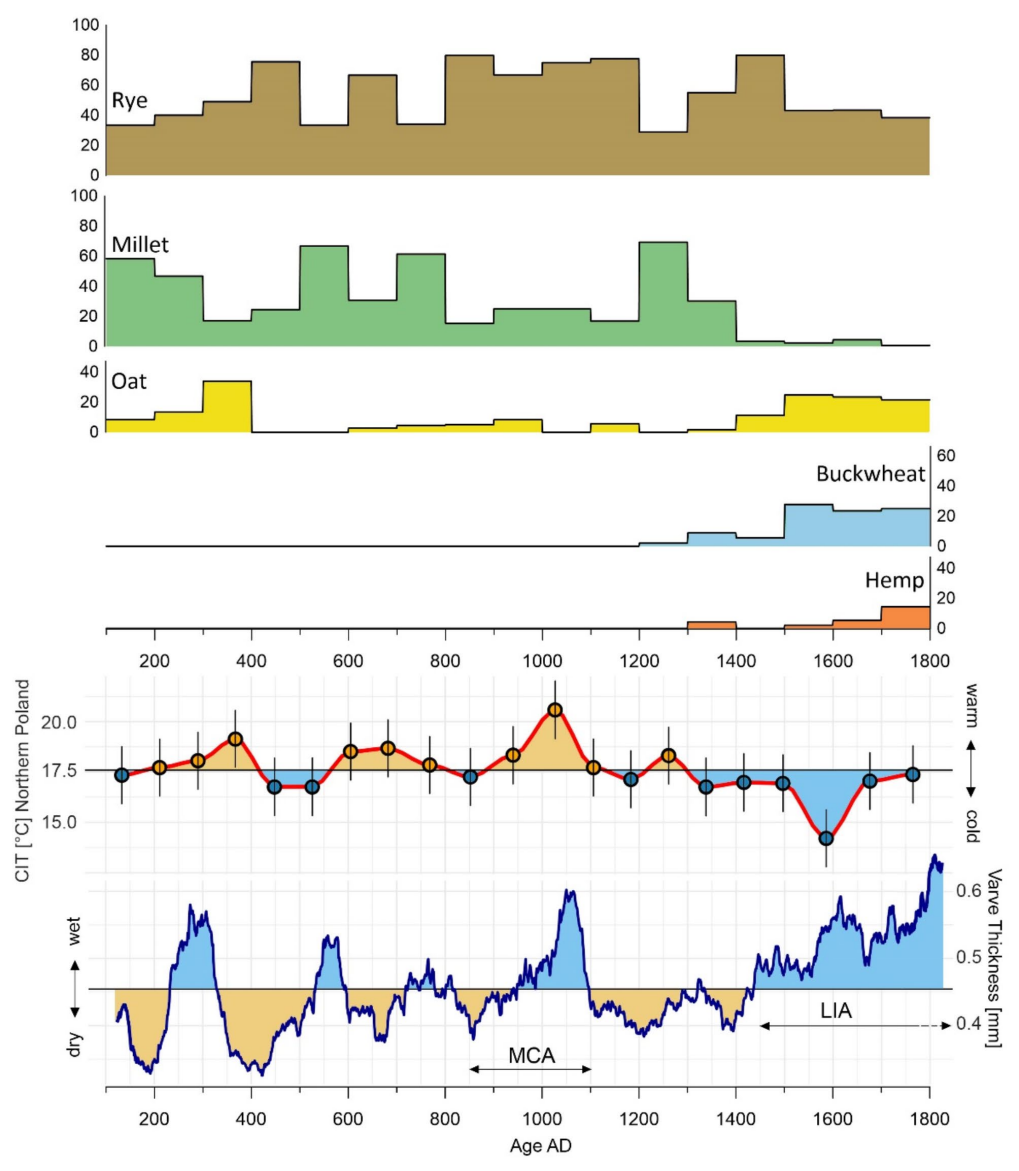
Chronological changes in cultivated crops (rye, millet, oat, buckwheat, and hemp) from 100 AD until the end of the 18th century, superimposed with general climatic trends. The CIT (Chironomid-Inferred Summer Temperature) reconstruction for Northern Poland (Lake Spore) is an indicator of growing season temperature variability in north-eastern Europe^24^. Varve thickness^25^ indicate wet or dry periods based on a lake sediment core from Central Finland.

Agricultural practices during 100–400 AD was significantly influenced by increasing temperatures and shifts in humidity, particularly after the first quarter of the 3rd century AD (Fig. 2). During the 2nd century AD, millet cultivation dominated as an adaptive response to drier environmental conditions. Spearman correlation test showed a positive correlation between millet and temperature (*r* = 0.25, *p* > 0.05) and a negative correlation with precipitation (*r* = -0.3, *p* > 0.05). Although not statistically significant, these trends reflect millet’s thermophilic nature and preference for drier conditions. However, starting from the 3rd century AD, rising temperatures and a shift in humidity around 300 AD led to a decrease in millet cultivation. While millet declined, rye and oats became prominent, with rye reaching approximately 50% of the crop repertoire and oats achieving their highest mean presence at 33.89% between 300 and 400 AD. This shift in crop distribution reflects the favorable climatic conditions that facilitated the success of rye and oats. Millet cultivation, however, dropped to as low as 20%, indicating changing agricultural priorities under these climatic conditions. The period from 400 to 900 AD presented significant agricultural challenges due to cooler and increasingly variable climatic conditions. During this time, rye and millet became critical crops, showing fluctuating dominance and opposing trends. Oats, however, were less prominent, disappearing entirely between 400 and 600 AD, likely due to their sensitivity to cooler conditions. Later, between 600 and 900 AD, oats reappeared with a low mean presence of around 4%, potentially reflecting a gradual increase in temperature. Spearman correlation test displayed a positive correlation between oats with precipitation (*r* = 0.3, *p* > 0.05) and no discernible correlation with temperature (*r* = 0, *p* > 0.05). This suggests that while oats may respond favourably to wetter conditions, their prevalence is likely influenced by other factors beyond climate alone.

Rye emerged as a dominant crop by the 5th century AD, reaching nearly 70% of the crop assemblage, coinciding with decreasing temperatures and dry conditions. Rye showed negative correlations with both precipitation (*r* = -0.25, *p* > 0.05) and temperature (*r* = - 0.05, *p* > 0.05). These weak correlations imply that rye, as a resilient staple crop, was less sensitive to climatic variability compared to other crops. Millet gained prominence during the 6th century AD, corresponding to wetter conditions and temperature fluctuations. A temporary increase in temperature and aridity between the mid-6th century and the 8th century AD saw millet cultivation rise, peaking at 61.36%. However, millet showed a declining trend by the 9th century AD, with rye becoming more prevalent and reaching mean percentages of almost 80%.

The period 900–1100 AD introduced generally warmer and wetter conditions, significantly shaping agricultural practices. During the 10th and 11th centuries, rye remained the dominant crop, with mean values around 25%. Oats showed a decline, disappearing entirely by the 11th century. The period 900–1100 AD introduced generally warmer and wetter conditions, significantly shaping agricultural practices (Fig. 2). During the 10th and 11th centuries, rye remained the dominant crop, with mean values around 25%. Oats showed a decline, disappearing entirely by the 11th century.

Between 1200 and 1300 AD, millet cultivation reached its peak, with a mean percentage of 69.23%, likely driven by a short rise in temperature and drier conditions around 1200 AD. This increase in millet cultivation contrasted with a decline in rye during the 13th century. The period also saw the first emergence of buckwheat, with a mean presence of 2.02%, reflecting its adaptability to evolving cooler climatic conditions. Buckwheat exhibited a statistically significant negative correlation with temperature (*r* = -0.5, *p* < 0.05) and a positive correlation with precipitation (*r* = 0.5, *p* < 0.05). This suggests that buckwheat thrived under cooler and wetter conditions, aligning with its increasing prominence during the LIA. Radiocarbon dating of two buckwheat grains confirmed their presence during this period. A sample from Vilnius, Arsenalo St. 5 [FTMC-DW62-1] has been dated to between 1286 and 1387 AD and a second from Maišiagala Hillfort [FTMC-DW62-2] to between 1276 and 1391 AD, representing the earliest evidence of buckwheat in the region.

The LIA (approximately around 1300–1800 AD)^26–29^ marked a shift to significantly colder conditions, which had profound effects on agricultural practices in the northernly latitudes. Between 1300 and 1400 AD, the climate was characterized by cold and dry conditions, particularly around 1400 AD. Rye remained a key staple crop during this time, although millet showed a drastic decline. As has been shown, the period between the fourteenth and fifteenth century AD saw the gradual increase in the cultivation of new buffer crops such as buckwheat and hemp as well as the recovery of oats.

By the beginning of the sixteenth century, buckwheat had reached its peak mean presence of 27.81%, highlighting its growing role as a buffer crop in response to the shift towards more humid and cooler conditions. Meanwhile, millet cultivation saw a further significant decline, with its mean values dropping to just 0.65% by 1700–1800 AD, indicating its reduced importance during wetter and colder periods. Oats recovered during 1500–1700 AD with mean values around 24%, also reflecting changes in crop selection due to the colder and wetter conditions. Additionally, hemp cultivation peaked at 14.43% between 1700 and 1800 AD, suggesting its increased use and importance during this time of agricultural adaptation to the harsher climatic conditions and the development of sea-faring industry. Hemp demonstrated a statistically significant positive correlation with precipitation (*r* = 0.5, *p* < 0.05) and a negative correlation with temperature (*r* = -0.5, *p* < 0.05). These correlations indicate hemp’s adaptability to wetter climatic conditions and its resilience to colder periods.

### Historical analysis

The systematic historical data we analyzed comes from the Early Modern period (approximately 1500–1800 AD). This period depicts the adaptive response of agriculture in the Grand Duchy of Lithuania to the climatic variability of the LIA, as well as an adaptation to new circumstances caused by transformation processes to novel farming models based on Central European patterns^30^. From the earliest period, we recorded 44 accounts detailing crop species cultivated on manors. In the 17th century, 60 accounts were documented, and by the 18th century, this number increased to 138 sources describing crop cultivation on large farms. All crop descriptions include information concerning what was sown on the farms. The term “sown” (original Polish “wysiane”) refers to the amount of grain allocated for planting in the fields during a particular year, measured in barrels or other units. Rye is mentioned 242 times while oats were also mentioned 233 times. Less frequently, the sowing of buckwheat was mentioned 203 times. Hemp and millet on large farms were sown less frequently. Millet was recorded in one third of all analyzed descriptions and mentioned 73 times, while hemp only 36 times.

**Fig. 3:**
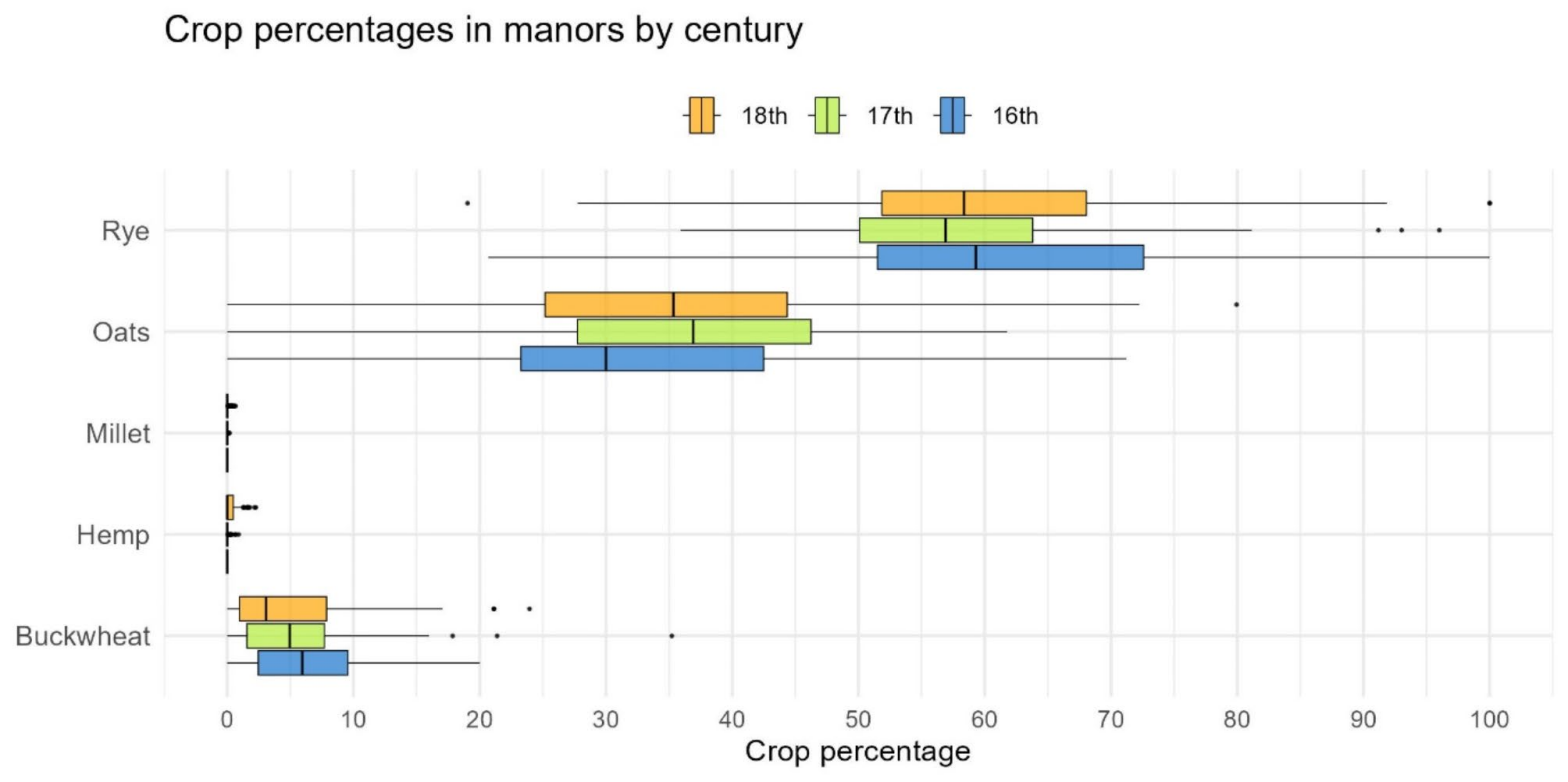
Percentages of sown crops in manors across the Grand Duchy of Lithuania during the 16th-18th centuries, as recorded in written sources.

The most important grain in the written records during the period between 1500 and 1800 AD was rye (Supplementary Dataset 3). It was noted in all archival sources. Among the cereals analyzed, more than 50% on average of the grain sown in the fields was rye (Fig. 3). It was mostly sown as a winter grain, but there were places where spring varieties of rye were sown. The share of rye among the analyzed cereals was very stable, with no significant changes seen over 300 years (p-value of the one-way ANOVA test = 0.867).

Similarly, recorded oat cultivation numbers were stable during the 16th to 18th centuries, and the seemingly smaller share of sown crops in the 16th century (30%) compared to subsequent centuries (above 35%) is not statistically significant (Kruskal-Wallis test p-value = 0.5445) (Fig. 3).

Buckwheat was far less frequently sown, but it was still an important food crop that was not exported but rather consumed locally. Its proportion of sown grain was modest, comprising only a few percent, and it gradually declined over time (although this trend is not statistically significant, with a p-value of 0.09). In the 16th century, buckwheat grain accounted for 6.5% of the observed cereals sown; in the following century its share fell below 6%, and in the 18th century below 5%.

Millet and hemp were not recorded in the inventories of the 16th century. In subsequent centuries, their share of sown grain was less than 1%.

**Table 1:** Values of rye, oats, millet, hemp, and buckwheat as described in the Third Statute of Grand Dutchy of Lithuania^31^ (in “Groschen”, which is the name for a silver coin used in the Grand Duchy of Lithuania.

	Rye	Oat	Hemp	Millet	Buckwheat
Value in Groschen of 1 Morgen* of Land Sown with Cereals	Winter rye: 180–300 Spring rye: 180	240	–	240	300
Value in Groschen of 60 sheafs* of Cereals	20	8	20	8	8
Value in Groschen of 1 barrel of Cereals	24	16	30	16	16

In the 16th century, one Groschen contained about 0.96 g of pure silver). “Morgen” is a historical unit of land measurement, whereas a sheaf denotes a bundle of grain stalks arranged lengthwise and bound together after harvesting.

Cereal values in the “Statutes” show the difference between the areas sown, the harvested, and the threshed crops^31,32^. The ratio of oat and millet are identical, for buckwheat, it differs only for the value of land sown. Meanwhile, the value of rye and hemp after harvesting is much higher. There are some reservations about this calculation because we must consider the difference in yields of different crops. Actually, on the estates of present-day eastern Lithuania in the 2nd half of the 16th century, buckwheat yield could reach 2.39–3.28 grains per unit, compared to 1.87–1.95 grains per unit for winter rye^33^. Here, “grains per unit” refers to the measure of cereal yield after harvest, where the quantity of grains initially sown is considered as 1; thus, a yield of 2.5 grains indicates 2.5 times the amount of grain originally sown. In addition, the differences in market value between different crops should be considered. For example, the value of hemp consists not only of the value of grains (for oil production) but also the value of the stem from which the fibre was made. The value of buckwheat consists of the value of grains and the value of the flowering field for honey production.

**Fig. 4:**
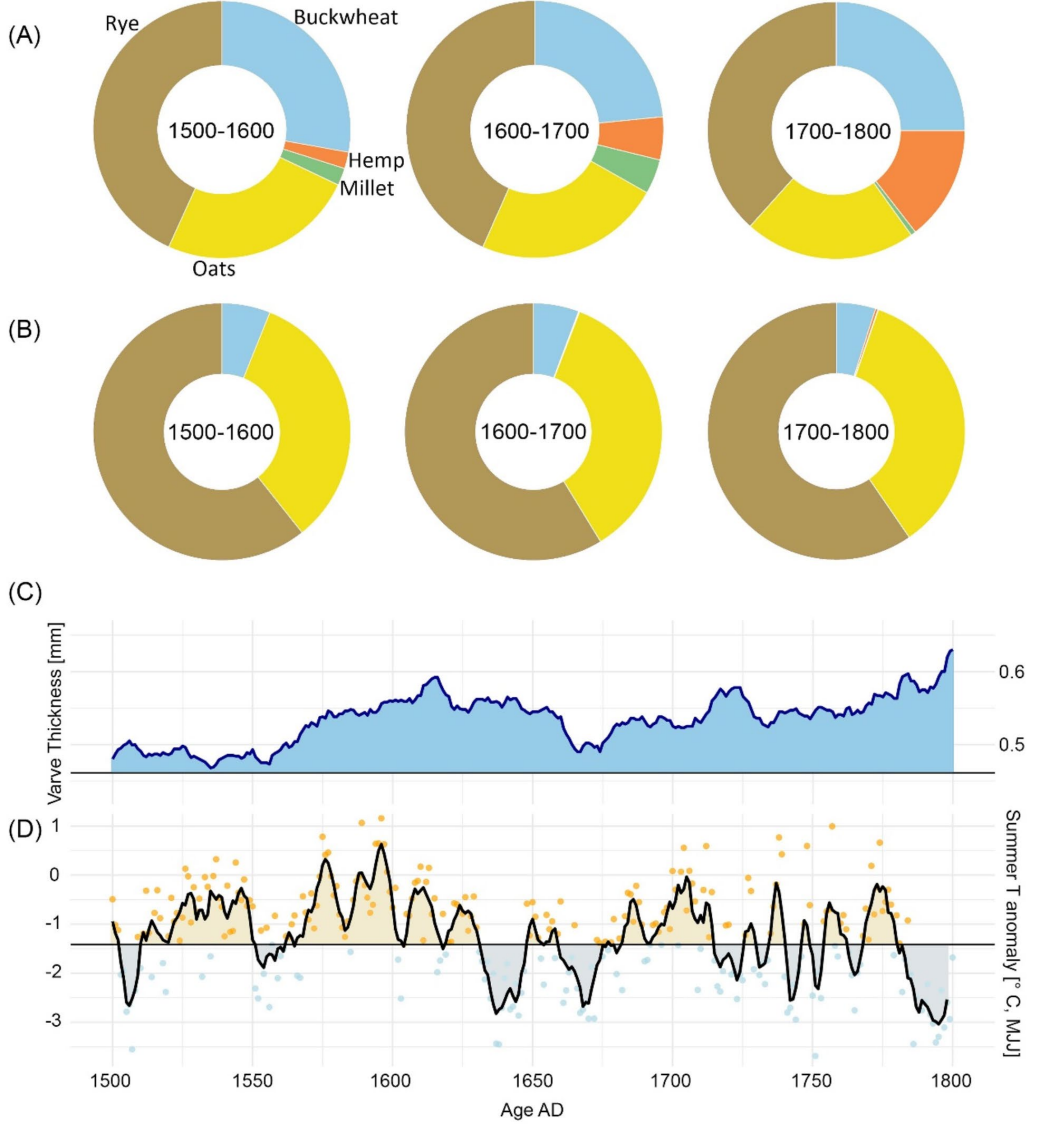
Crop distribution from archaeobotanical and historical records in relation to paleoclimatic reconstructions: (**a**) Pie charts showing the crop distribution from archaeobotanical data across three temporal bins (1500–1600, 1600–1700, 1700–1800). The distribution includes crops like rye, oats, buckwheat, hemp, and millet. (**b**) Pie charts representing crop distribution derived from historical records over the same temporal bins (1500–1600, 1600–1700, 1700–1800). (**c**) Varve thickness record from the Central Finland lake as indicator of wet/humid conditions^25^. (**d**) May-July temperature anomaly for Eastern Europe, indicating periods of warm/cold conditions^1,38^.

The crop distribution data from archaeobotanical sources (Fig. 4a) demonstrate notable changes from 1500 to 1800, with a gradual shift in the proportions of crops like buckwheat, hemp, and millet relative to rye and oats. In contrast, the historical records (Fig. 4b) indicate relatively stable crop distribution patterns over the same period. This suggests that while historical accounts reflect consistent agricultural practices, the archaeobotanical evidence points to a more dynamic response to the colder and wetter conditions indicated by the paleoclimatic reconstructions (Fig. 4c and d), particularly during the 17th century.

## Discussion

It is striking that over the whole period of interest (100–1800 AD), the proportion of the five crops investigated in this study not only varied considerably but also alternated over time, showing a pattern of crop replacement over the centuries (Fig. 2). Importantly, these changes followed changes in climatic conditions. This section discusses the above-described results in a diachronic perspective.

### Focus on the pre-LIA period (100–1300 AD)

Starting with the two dominant crops, rye and millet, they seem to take turns depending on temperature and to a lesser extent on humidity. Hence, the proportion of millet is usually increasing when temperature increases and humidity decreases, which is to be expected since millet is a thermophilic and drought-resistant plant that has a rapid but limited growing season^17^. This suggests that this crop could better adapt to the typically short summers in northern latitudes provided that the growing conditions were suitable. In other circumstances, rye, mainly a winter crop, favouring mild winters and poor soils^39^, became dominant again. Hence, despite the elevated temperatures during the period between around 900 and 1100 AD, the millet proportion expectantly increased compared to the previous and subsequent cooler centuries, yet rye remained the predominant crop, as moist climate prevailed. The only important exceptions to this pattern are to be found in the 4th and 7th century AD, when millet proportion decreased sensibly despite warm and dry conditions. These trends could be connected to cultural transformations and the delay to adjust agricultural practices to climate change, or simply a result from the sample size and the potentially biased chronological resolution related to the chronological bins. Overall, millet nevertheless demonstrated resilience, suggesting that its cultivation remained viable even under cooler and to some extent wetter conditions (Fig. 2). Agronomical records from the early 17th to late 18th centuries support millet’s adaptation to changing climatic conditions during cold and wet spells indicating shifts in the sowing times of millet in present day Poland, moving from April or March to May or June as the growing season was reduced^17^. This shows that agricultural practices were dynamic and provided adapted responses to climatic variability by, for example, buffering the rye production in periods of drought, and by offering more diversity that enabled to effectively cope with climatic instability.

In the first medieval description of agriculture in Central Europe, which comes from the traveler Ibrahim ibn Ya‘qub during his stay in Bohemia in the second half of the 10th century AD, it is written about the Slavs that “They sow in two seasons, in summer and in spring, and harvest two crops”^40^. Historians dealing with the history of agriculture in Central and Eastern Europe agree that at the time of the birth of the first states (10th century AD), the arable farming at Slavs and Balts functioned in a two-field system. Under this system, rye was the main winter cereal, and millet was the main spring cereal^41^. More developed - the three-field system, originally applied in the Carolingian empire in the 8th and 9th centuries AD, spread in central Europe with German colonization^30^. It triggered a change in the late medieval structure of agricultural production. This was especially true for spring cereals, among which oats became the most important^42^, as will be discussed below.

### Focus on the LIA and its onset (1300–1800 AD)

The most obvious observation is, however, represented by the increase in hemp, buckwheat, and oat cultivation from around 1200 AD onward corelated with significant decrease in millet cultivation, which coincides with a temperature decline and a sharp increase in humidity (Fig. 2). In fact, the particularly wet conditions of the LIA recorded from around 1450 AD notably favoured the cultivation of these plants at the expense of millet, despite millet’s long tradition in the region of present-day Lithuania. Archaeobotanical data from countries in close geographical proximity to the south, including Poland^43,44^, the Czech Republic^45^, and Germany^46–48^, attest to the continued consumption and cultivation, of millet during the 15th-18th centuries. As the northern limit of millet cultivation had shifted further south, the exceptionally scarce remains of millet discovered above 54°N latitude may be attributed to commercial imports, as it appears Hanseatic exchange records, such as those from Tartu^49^.

**Fig. 5:**
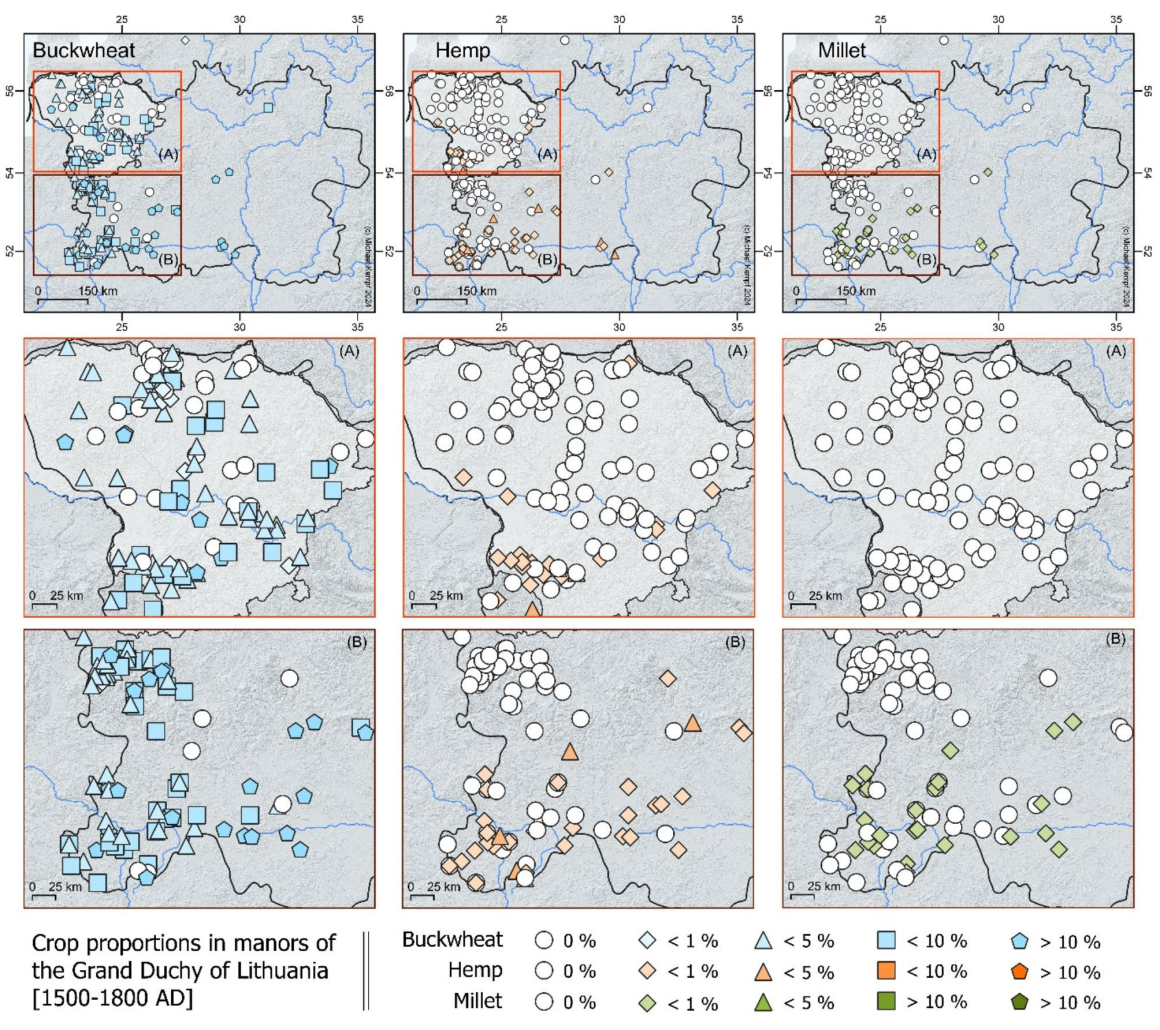
Buckwheat, millet and hemp in the manors in the Grand Duchy of Lithuania (1500–1800 AD). Modern Lithuania is highlighted as white shade. This map is produced using QGIS 3.10.12 (QGIS Geographic Information System. QGIS Association. http://www.qgis.org (2024)).

As supported by historical data, millet being sown increased progressively further south within the Grand Duchy of Lithuania, where buckwheat also accounted for approximately 10% of the cultivated fields. The historical sources indicated that millet and the groats made from it still played a key role in the diet of 14th century southern regions of Poland, including that of representatives of the elite. The Polish King and Grand Duke of Lithuania Ladislaus Jagiello, who served millet to his guests at his residency in Cracow of southern Poland^50^. An analysis of the written sources on the gastronomic culture between the 16th and 18th centuries^51^ shows that buckwheat replaced millet in the food structure of the lower social classes in the regions to the north of present-day Lithuania. Meanwhile, rice partly replaced millet in the diet of the higher social classes. It is known that groats made from millet and buckwheat (as well as barley, which goes beyond the scope of this study) played an important role in the diet of the inhabitants of the Polish-Lithuanian state^17,52,53^. In this context, buckwheat was grown throughout the Grand Duchy of Lithuania (in what is now Lithuania and Belarus), while millet was found in the southern and southeastern regions of this historic country (Fig. 5).

In parallel, after 1300 AD botanical remains show that the proportion of rye decreased sensibly, yet it remained the dominating crop as demonstrated by both the archaeobotanical and historical sources of this period. Rye was not only the main bread grain but also the main cereal product exported from the Polish-Lithuanian Commonwealth throughout the period (1500–1800 AD). The second economically most relevant cereal remained oats, a staple of animal feed on farms^54^. And again, an alternation pattern is visible, as in the late medieval and early modern agricultural system, rye was the most important winter cereal, while oats were among the most essential spring cereals. These two staple cereals, whose decisive role between 1500 and 1800 AD is unanimously confirmed by historical and archaeobotanical materials, were supplemented by other plants relevant to human and animal consumption. Indeed, buckwheat, hemp and oats are known for their resistance to cold and moist conditions^55–57^ and the expanded use of these buffer crops was necessary to diversify agricultural strategies and adapt to the increasingly humid conditions of the LIA.

Finally, the rise in hemp cultivation is attested both historically (Fig. 5) and archaeologically. This could be linked with its ability to cope with climate variability, as the discovery of hemp fruits in many domestic spaces is associated with food preparation. Also, north-eastern Baltic written sources from the 16th-19th century depict hemp as an important source for human food along with valuable fibre. The main food product being hemp seed oil^36^, while hemp seed soup^36^, flatbread^37^ and a stew-style dish called “stakanis”^34^ are also attested. Meanwhile, the growth in hemp production from the 14th century onward may have been mostly driven by the increased demand for hemp fibre during the era of the Great Geographical Discoveries and the subsequent development of global shipping, in which the Baltic played an important role. In this framework, hemp constituted a vital component for shipping materials and played together with rye and wood an important role in exports from the Lithuanian Grand Duchy^58^. Based on the written sources, it can further be assumed that hemp was essentially cultivated on market-oriented farms, especially those produced for export.

## Conclusions

This archaeobotanical and historical analysis reveals that crop cultivation in northeastern Europe between 100 and 1800 AD was notably influenced by climatic changes and socioeconomic factors. The observed trends in rye, oat, millet, buckwheat, and hemp cultivation highlight the adaptability and resilience of agricultural practices in response to climatic changes. In particular, buckwheat and hemp proportions show statistically significant correlation with climatic variables, while rye remained the dominant crop throughout the studied period, demonstrating resilience to a range of climatic conditions. Its persistent presence as reflected by both archaeobotanical and historical records underscores its importance as a staple grain.

Millet, with its thermophilic and drought-resistant properties, thrived during periods of increased temperature and drier conditions, such as 200–300 AD, 700–800 AD, and 1200–1300 AD. However, its cultivation declined during the cooler and wetter LIA after the 14th century, with only sporadic evidence of its presence in the historical Grand Dutchy of Lithuania. Historical sources further support this shift, showing that the northern limit of millet cultivation during the period 1500–1800 AD period had moved progressively further south. In contrast, cold-resistant crops such as rye, buckwheat, oat and hemp played an essential role in the prompt reorganization of agricultural systems to mitigate vulnerability to these harsher conditions.

However, historical data suggest that socioeconomic factors, including trade and evolving dietary preferences, also played a pivotal role in shaping agricultural practices, particularly during the Late Medieval and Early Modern period. Hence, hemp cultivation likely increased due to its dual utility as a buffer crop and an essential raw material for the maritime trade. These findings provide valuable insights into the historical interplay between climate, agriculture, and human adaptation.

Future climate predictions suggest that a shift to crops like millet might be necessary for successfully tackling climate change. Understanding historical trends can inform modern agricultural strategies to enhance resilience and sustainability in the face of ongoing climatic challenges.

## Materials and methods

### Paleoclimatic overview of the study area

The study area includes Lithuania, eastern Poland, and part of the Belarus territory approximately from ca 52° N to 58° N in latitude and from 20°57’18’’ E to 32°47’00’’ E in longitude (Fig. 1) of semi-continental climate that, unlike western Scandinavia of the same latitude, is not affected by the Gulf Steam-influence. In the Eastern Baltic region, the minor shifts in seasonal and vernalization patterns could significantly influence plant development and growth^59^.

The research area is at the intersection of the Eurasian continental and oceanic western European climate zones. The landscape and the environment in the Eastern Baltic were significantly shaped during the last Ice Age^60–63^. The Northern Atlantic serves as the primary moisture source in this region^26^. The Eastern Baltic region, characterized by a humid continental climate (*Dfb*) under the Köppen climate classification, exhibits an average annual temperature of approximately 6 °C. Seasonal temperature variations are notable, with winter temperatures averaging − 4.4 °C and summer temperatures averaging 16.3 °C. Minor variations between coastal and continental areas reflect the maritime and continental influences of the region. Annual precipitation averages around 692 mm, with differences between coastal and continental zones^64^. Importantly, precipitation generally exceeds evapotranspiration in this region, with a ratio of 1.47^65,66^.

The climatic history of the region, spanning the last two millennia, has been extensively studied using various paleoclimatic proxies, including pollen, geochemical data, Cladocera and chironomid assemblages, tree-ring data, macrofossils, testate amoebae, Mg/Ca ratios, and isotopic compositions (δ13C, δ18O), which were combined to Supplementary dataset (S2). These data were derived from sedimentary archives such as lakes, peat bogs, terrestrial landscapes, speleothems, and tree ring records within northeastern Europe and adjacent areas (Supplementary Dataset 2).

The last two millennia witnessed significant climatic fluctuations, which are categorized into four major climatic periods (Supplementary Dataset 2). The major regional climatic trends were recorded in northeastern Europe represent:

- Medieval Climatic Anomaly: warm and mild with cold spells from ca. 900 AD to 1300 AD^26–30^.
- Little Ice Age: colder temperatures from around 1300 to 1800 AD, marked by significant variability in moisture level^28,29,35,67,68^. For the study region the period before 1500 AD is characterized by dry and after 1500 AD by wetter conditions^24,35,68^.

### Buffer crop concept

A buffer crop is defined as a crop that mitigates risks associated with adverse climatic or environmental stress, providing stability to agricultural systems during periods of instability. Buffer crops are particularly valuable in regions or periods prone to unpredictable weather patterns, such as droughts, cold spells, or soil degradation. Their cultivation is an adaptive strategy that ensures agricultural productivity under challenging conditions^9–11^.

In this study, rye, millet, oat, hemp, and buckwheat function as buffer crops due to their resilience to a variety of stressors, including drought, cold climates, and poor soil quality. These crops have historically been integral to farming systems in regions subject to climatic variability or poor soils, offering a form of insurance against crop failure.

### Data sources and selection

Primary archaeobotanical data for this study was obtained from 135 contexts across 98 archaeological sites, with 60 of these sites represented by samples stored and analyzed at the Bioarchaeology Research Centre of Vilnius University since 2014 (Supplementary Dataset 1). The remaining 38 sites were integrated from archaeological reports (after reviewing over 2000 of them published until 2024) and articles from various Lithuanian excavations, following the criteria established for this study. Detailed data on the distribution of sites across temporal bins from 100 to 1800 AD and different climatic periods are presented in the Supplementary dataset (S1). The initial temporal bin from 1 to 100 AD was excluded due to its limited representation; only one site was identified where a single grain of rye was discovered.

The analyzed plants were recovered from archaeological excavations as charred, waterlogged, or mineralized remains. Charred materials, particularly those subjected to carbonization through burning, maintain identifiable morphological characteristics. The organic matter in these materials is converted to inert carbon, which preserves their physical structure and ensures their chemical stability^69^. Waterlogged remains were preserved in wet and anoxic environments, while mineralized remains were typically found in cesspits. For consistency, we only used plants that could be identified at the species level. We have focused on five crop species Oat (*Avena sativa*), Rye (*Secale cereale*), Millet (*Panicum miliaceum*), Buckwheat (*Fagopyrum esculentum*), Hemp (*Cannabis sativa*). These buffer or marginal crops are the most relevant from the perspective of agricultural adaptation to climate change. Also, at this stage, it is important to emphasize that millet, rye, oat and the other crops are not equally represented in the archaeobotanical context depending on the preservation conditions at the archaeological site and sample collection strategies. Wheat was excluded from this study as in many archaeobotanical and historical sources it is referred only to genus and not to species. There are around twenty species of wheat with varying ecologies and with the absence of archaeobotanical chaff remains wheat identification to species became unsecure. Future studies should address wheat separately, with detailed species identification, to provide a more nuanced understanding of its historical distribution and significance.

In this study, we also excluded barley as botanical reports or historical data do not always specify barley varieties that are indicative of this crop’s resilience and adaptation to varying environments^70^. Its diachronic change from compact naked six-row forms to two-row hulled forms indicated adaptation to changing environmental conditions in Central Asian mountains^71^. Past studies have shown a change from naked barley varieties in Bronze Age Europe to hulled ones in later periods^72^ that could have been influenced by both cultural and climatic factors, while the rise of hulled two-row varieties in the Early Modern period was due to the development of brewing industry in the Eastern Baltic. Therefore, without the specificity of the barley variety, the integration of barley into the dataset would not be sufficiently informative and could introduce some biases toward the results of other minor crops. The occurrence of *Camelina sativa*, *Linum usitatissimum*, *Vicia faba* or *Pisum sativum* is also very sporadic and rare in broader archaeobotanical contexts, therefore we did not include those crops in our datasets.

Temporal bins of 100 years were used to categorize archaeobotanical data points into distinct time intervals (Supplementary Dataset 1). We defined the size of each temporal bin as 100–200 AD, 200–300 AD, and so forth. To assign each archaeobotanical site to its corresponding temporal bin, we used the midpoint between the earliest and latest dates. However, sites with excessively wide chronological ranges were excluded to ensure temporal accuracy, as some materials spanned several centuries and could not be reliably placed within narrower 100-year bins. The next step involved aggregating the crop percentages for each temporal bin and summarizing the data within each bin to facilitate subsequent analysis and interpretation.

### Historical sources

Systematic historical sources on different crop cultivation in Grant Dutchy of Lithuania only date back to the 16th century. These are the inventories of manors i.e., large agricultural and livestock farms. Inventories were the accounting documents. They usually recorded the buildings of the manor, the movable property, the people who worked on the manor, the livestock, the crops, the manufacturing enterprises, etc. For this study, we analyzed historical sources from the early modern period (1500–1800 AD), that contain 242 descriptions of the structure of crops on the manors owned by the king or representatives of noble families in the Grand Duchy of Lithuania (part of the Polish-Lithuanian Commonwealth) (Fig. 1).

Some of the documents used for this study have been published, but most remain in manuscripts, which are held in archives in Poland (The Central Archives of Historical Records in Warsaw), Lithuania (Lithuanian State Historical Archives in Vilnius), and Belarus (National Historical Archives of Belarus in Minsk). The data we collected came from the territories of modern eastern Poland, Latvia, Lithuania, and Belarus. The manor inventories usually provide information on the amount of grain sown (less often harvested). In our analysis, we determined the relative proportions of the various crops, paying particular attention to rye, which was the main bread grain and shipped for export along with oats, which was the primary animal feed (used to a lesser extent in human consumption), as well as millet, hemp, and buckwheat. The results obtained are representative primarily of large agricultural producers, but because of the way fields are organized and the use of peasant labor under the serfdom system, they also largely correspond to the peculiarities of ordinary peasant farms.

The integration of historical sources with archaeobotanical data provides valuable insights, but several limitations must be considered. The structure of sown crops does not fully represent the harvested crop structure or the relative importance of individual cereals. Variations in sowing density and yield across different cereals suggest that the actual significance of crops like hemp and millet may have been greater than what is indicated by their sowing proportions. While our analysis focuses on five crops: rye, oats, millet, hemp, and buckwheat, it is important to note that wheat and barley also contributed significantly to crop production, particularly with the growth of the brewing industry in the 18th and 19th centuries. Together, wheat (species is not specified) and barley accounted for 10% and 20% of the total area sown in the manorial fields, varying according to geographical location and market proximity. These reservations could be partially clarified by comparison of the data on the values of different cereals found in legal documents such as the Third Statute of the Grand Duchy of Lithuania (1588). The Statute refers to cereals in three ways: in the calculation of the value of fields sown with cereals, in the valuation of cereals already harvested (before threshing), and in the valuation of pure grains (after threshing) (see Table 1).

From the written sources, it was possible to determine the percentage of the five investigated crops sown at manors from the research area within the 16th, 17th and 18th centuries. To assess whether the observed changes over time were statistically significant, we applied a one-way ANOVA test for the normally distributed crops (rye) and a Kruskal-Wallis test for the other crops (oats, hemp, buckwheat and millet). The code is provided in the Zenodo repository and comprises the results of each test^73^.

### Climate data evaluation and testing strategies

All statistical analysis, tests, and modelling was performed using R software^20^. R-code and data to replicate the results can be accessed via this Zenodo repository^73^ 10.5281/zenodo.14175503. We are testing climate variability in the study area against crop distribution and development patterns during the period 100–1800 AD with a particular focus on the pre-industrial modern era (1500–1800 AD)^73^. Because eastern European climate reconstructions are sparse, we deploy a varve lake sediment record, which is an indicator for humid or dry conditions deriving from central Finland lake deposits^25^. Large-scale climate systems can show local and regional variations, which is why we tested the spatial correlation of precipitation patterns at the sample location region and the study area of this paper. Monthly precipitation data was accessed using the Koninklijk Nederlands Meteorologisch Instituut climate explorer (https://climexp.knmi.nl/allstationsform.cgi?id=someone@somewhere, last accessed 17th of November 2024). We selected 15 near-by stations for each of the regions and calculated the mean annual precipitation records to avoid missing values during, e.g., WWI and WWII. The data spans from 1891 to 2017 and was tested for correlation to detect similar spatial behavior of precipitation variability of the long-term time series. We find that both time series correlate well (corr 0.82036878028) after approximately 1930 (see repository for scatterplots and cubic spline smoothed time series plots). We conclude that the central Finland sample location is also representative for Lithuanian precipitation patterns during summer months growing season. The varve record was smoothed with a rolling 50 year average smoothing operation using the entire span of the available record. For temperature anomaly reconstructions for the chronological subset of the 16th to the end of the 18th century, we accessed the data provided by Ulf Büntgen and colleagues^1,38^, which is spatially consistent with our study area but does not span the entire period under consideration for this article. Hence, for the entire comparison period 100–1800 AD, we used the Chironomid inferred summer temperature reconstruction (CIT)^24^ as a proxy for pre-modern temperature variation across the study area, binned to 100-year bins. The analysis aimed to assess the general relationship between crop development, represented by the appearance of different crops (oat, rye, millet, buckwheat, and hemp) and climate variables (wetness/drought indicator from the Finish lake sediments; temperature reconstruction from chironomid records of Lake Spore Northern Poland^24^) over a historical time span, using a permutation test (*n* = 10.000, see code and repository to this article). The data was organized by century, with crop appearance expressed as percentages for each type and climate variables binned to the same periods. To quantify the relationship between crop appearance and climate variables, a Spearman’s rank correlation was calculated. Due to the sample size and the palaeobotanically forced bin size, the non-parametric measure of correlation was chosen because it assesses monotonic relationships without assuming a linear or normally distributed relationship between the variables. The permutation test was employed to assess the significance of the observed correlation. This involved calculating the observed Spearman correlation between the crop appearance and the shuffled climatic variables, recalculating the correlation for each permutation to create a null distribution. Eventually, we compared the observed correlation to the null distribution to determine the likelihood of the observed correlation occurring randomly (see code to the paper).

## Electronic supplementary material

Below is the link to the electronic supplementary material.

Supplementary Material 1

Supplementary Material 2

Supplementary Material 3

## Data Availability

We provided all data reported in the article in Supplementary Information files and in this repository: Kempf, M. (2024). Code and data to “The shifting of buffer crop repertoires in pre-industrial north-eastern Europe " [Data set]. In Scientific Reports. Zenodo. https://doi.org/10.5281/zenodo.14175503 Supplementary Information:1. Supplementary Dataset 1.Supplementary Dataset 1 contains archaeobotanical data and radiocarbon dates.2. Supplementary Dataset 2. Overview of paleoclimate data.3. Supplementary Dataset 3. Historical dataset from manors.

## Declarations

### ^14^C Dating

A radiocarbon dating of two charred buckwheat (*Fagopyrum esculentum*) seeds was performed (Supplementary Dataset 1 (S1)). The first buckwheat seed [FTMC-DW62-1] from Vilnius’s Arsenalo St. 5 is estimated to have originated in the years 1286–1387 AD (95.4% probability). Dating between 1276 and 1391 cal. AD (95.4% probability) is the second buckwheat seed [FTMC-DW62-2] from Maišiagala Hillfort.
