# Correction: The shifting of buffer crop repertoires in pre-industrial north-eastern Europe

**DOI:** 10.1038/s41598-025-22314-6

**Published:** 2025-10-06

**Authors:** Meiirzhan Abdrakhmanov, Michael Kempf, Ruta Karaliute, Piotr Guzowski, Rimvydas Lauzikas, Margaux L. C. Depaermentier, Radosław Poniat, Giedre Motuzaite Matuzeviciute

**Affiliations:** 1 Department of Archaeology, Faculty of History, Vilnius University, Universiteto St. 7, 01513 Vilnius, Lithuania; 2 Quaternary Geology, Department of Environmental Sciences, University of Basel, Bernoullistrasse 32, 4056 Basel, Switzerland; 3 Department of Geography, University of Cambridge, Cambridge, UK; 4 Lithuanian Institute of History, Tilto St. 17, 01101 Vilnius, Lithuania; 5 Faculty of Communication, Vilnius University, Saulėtekio Ave. 9, 10222 Vilnius, Lithuania; 6 Faculty of History, University of Bialystok, Plac NZS 1, 15-420 Bialystok, Poland

Correction to: *Scientific Reports* 10.1038/s41598-025-87792-0, published online 29 January 2025

In the original version of this Article Giedrė Motuzaite Matuzeviciute was omitted as a corresponding author. Correspondence and requests for materials should also be addressed to giedre.keen@if.vu.lt.

The original Article has been corrected.

